# Neuronal activity triggers uptake of hematopoietic extracellular vesicles in vivo

**DOI:** 10.1371/journal.pbio.3000643

**Published:** 2020-03-16

**Authors:** Ivan-Maximiliano Kur, Pierre-Hugues Prouvot, Ting Fu, Wei Fan, Felicia Müller-Braun, Avash Das, Saumya Das, Thomas Deller, Jochen Roeper, Albrecht Stroh, Stefan Momma

**Affiliations:** 1 Institute of Neurology (Edinger Institute), University Hospital, Goethe University, Frankfurt am Main, Germany; 2 Institute of Pathophysiology, University Medical Center of the Johannes Gutenberg University, Mainz, Germany; 3 Leibniz Institute for Resilience Research, Mainz, Germany; 4 Institute of Neurophysiology, Neuroscience Center, Goethe University, Frankfurt am Main, Germany; 5 Cardiovascular Research Center, Massachusetts General Hospital, Boston, Massachusetts, United States of America; 6 Institute of Clinical Neuroanatomy, Neuroscience Center, Goethe University, Frankfurt am Main, Germany; Baylor College of Medicine, UNITED STATES

## Abstract

Communication with the hematopoietic system is a vital component of regulating brain function in health and disease. Traditionally, the major routes considered for this neuroimmune communication are by individual molecules such as cytokines carried by blood, by neural transmission, or, in more severe pathologies, by the entry of peripheral immune cells into the brain. In addition, functional mRNA from peripheral blood can be directly transferred to neurons via extracellular vesicles (EVs), but the parameters that determine their uptake are unknown. Using varied animal models that stimulate neuronal activity by peripheral inflammation, optogenetics, and selective proteasome inhibition of dopaminergic (DA) neurons, we show that the transfer of EVs from blood is triggered by neuronal activity in vivo. Importantly, this transfer occurs not only in pathological stimulation but also by neuronal activation caused by the physiological stimulus of novel object placement. This discovery suggests a continuous role of EVs under pathological conditions as well as during routine cognitive tasks in the healthy brain.

## Introduction

Extracellular vesicles (EVs) emerge as ubiquitous signaling agents that can transfer functional proteins, nucleic acids, and lipids between cells in vitro and in vivo [1–4]. The ability to transfer such a range of different molecules is particularly intriguing in the communication between the brain and the periphery. Moreover, EVs can contain signature molecules that may reflect the physiological state of secreting neural cells, passing the blood–brain barrier (BBB) and thus making them obvious targets for diagnostic use. Vice versa, EVs may be a valuable tool for the delivery of therapeutic molecules to the brain [5,6]. However, the physiological parameters that regulate EV signaling to the brain in vivo are poorly understood. Here, we investigate whether the uptake of EVs released by peripheral blood cells is triggered by neuronal activity. In order to track EV signaling in vivo, we used a transgenic mouse model developed by us that can be leveraged to trace the transfer of functional mRNA by blood-derived EVs in vivo using the Cre-LoxP recombinase system [2,3]. Our results demonstrate that peripheral inflammation induced by injection of lipopolysaccharide (LPS) leads to a massive onset of blood-to-brain signaling via EVs. We made similar observations in models that are more or exclusively characterized by neuronal activation such as kainate (KA) injection, pharmacological inhibition of the proteasome in dopaminergic (DA) neurons, and, most specifically, optogenetic induction of neuronal activity. Furthermore, neuronal activity and concomitant EV uptake can also be induced by behavioral cues as in novel object placement in vivo. Together, this argues for a continuous communication of blood to brain via a transfer of functional molecules contained in EVs in various pathological and physiological states. The transfer of molecules by EVs that were so far not considered in intercellular signaling may be of great importance [1].

## Results

### LPS-induced peripheral inflammation leads to widespread EV uptake in the brain

**Fig 1:**
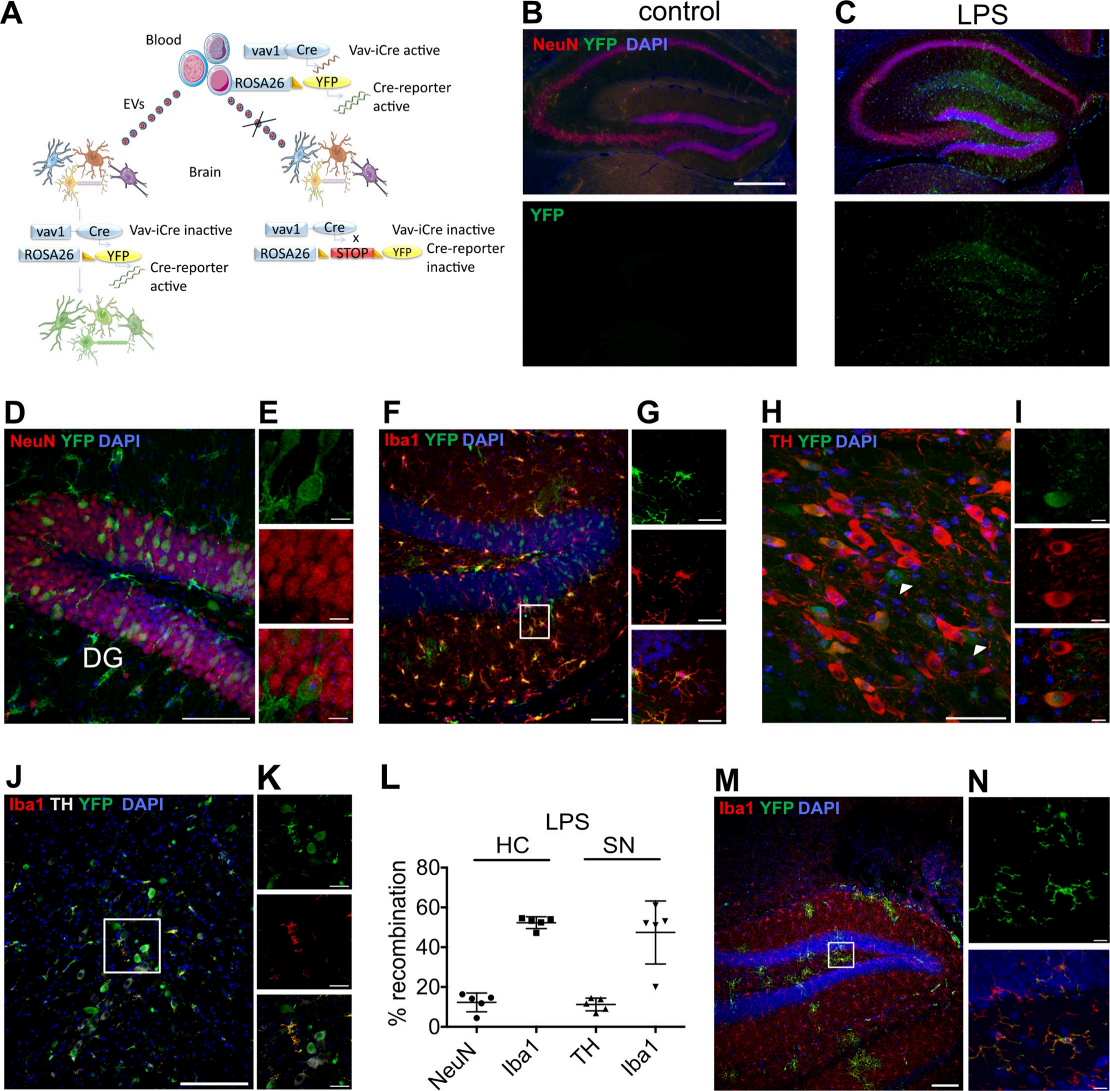
Peripheral stimulation initiates widespread EV uptake in the brain. (A) Schematic representation of the method to map EV mRNA transfer from blood to the brain. Cre mRNA contained in blood-derived EVs is taken up by neural cells, leading to excision of the stop-loxP site and induction of marker gene expression. (B) HC from a control vav-iCre-R26EYFP mouse compared to 48 h after IP LPS injection showing frequent recombination events in the hippocampal area (C–G). Recombined cells are predominantly neurons and microglia (D–G), including DA neurons (H + I) and microglia (J + K) in the SN. Arrowheads indicate recombined cells with a neuronal morphology that are TH-negative. Both structures show similar levels of recombination. (L) Data are presented as mean ± SD. *p* = 0.000666 two-tailed nonparametric Wilcoxon–Mann–Whitney U test for all populations compared to zero marker-gene–positive cells in all control animals, *n* = 10, underlying data can be found in S2 Table. (M + N) Recombination after LPS treatment in EpoR-iCre mice is restricted to microglia. Scale bars, 500 μm in B; 100 μm in D, F, J, M; 10 μm in E, G, I; 50 μm in H; 25 μm in K; 5 μm in N. DA, dopaminergic; DG, dentate gyrus; EV, extracellular vesicle; EYFP, enhanced yellow fluorescent protein; HC, hippocampus; IP, intraperitoneal; LPS, lipopolysaccharide; SN, substantia nigra; TH, tyrosine hydroxylase; YFP, yellow fluorescent protein.

The transgenic mouse model that we were using for our study relies on the fact that Cre mRNA is expressed in hematopoietic cells under the vav1 promoter [7] and sorted into EVs that are then released into the bloodstream. The exact mechanism of how the Cre mRNA is sorted into exosomes is unclear, but it is probably a simple reflection of high cellular expression [8]. Upon entering a target cell, Cre mRNA is translated to functional protein, leading to the irreversible onset of a marker gene expression, here enhanced yellow fluorescent protein (EYFP), mediated by Cre recombinase activity (Fig 1A). In the healthy animal, no or very few recombined neural cells that are restricted to Purkinje neurons in the cerebellum indicative of EV signaling are present in the brain [2,9] (hippocampus [HC] in Fig 1B). However, after peripheral inflammation by 2 daily intraperitoneal (IP) injections of LPS (1 mg/Kg), we observe frequent recombination events in the HC (Fig 1C to 1G) and substantia nigra (SN) (Fig 1H to 1K) but also in other brain regions (S1 Fig). For the dentate gyrus (DG) of the HC, marker gene NeuN-positive neurons reached an average of 12.3% (±4.7% SD, *n* = 5), and Iba1-positive microglia reached 52.3% (±3% SD, *n* = 5) (Fig 1L). In addition, we observed 11.2% (±3.2% SD, *n* = 5) of tyrosine hydroxylase (TH)-positive DA neurons expressing EYFP and 47.4% of Iba1-positive microglia (±15.8% SD, *n* = 5) in the SN (Fig 1L). For the SN, we could also frequently observe recombined cells with neuronal morphology that were TH-negative (white arrows in Fig 1H). Given the prominent role of inflammation in Parkinson’s disease, this may point to a previously unidentified factor linking these events. We very rarely or never observed cells of the endothelial (CD31), astrocytic (glial fibrillary acidic protein, GFAP), or oligodendroglial (Olig2) lineage coexpressing the marker gene (*n* = 3 mice) (S2 Fig). As a complementary experiment to our vav-iCre mice and in order to gain further insight into the role of the origin of EVs in the selectivity of cell targets, we analyzed brains from erythropoietin receptor (EpoR)-iCre mice (*n* = 3) after peripheral LPS injection. In EpoR-iCre mice, iCre expression is restricted to the erythroid lineage [10]. Analysis of brain sections revealed the absence of neuronal marker gene expression while still observing recombined Iba1-positive microglia (Fig 1M and 1N), indicating that the targeting of EVs to specific cells is determined, at least in part, by the cell of origin.

To probe for more specific stimulation of neuronal activity we injected KA, a neuroexcitatory agonist for KA receptors that is frequently used as an epilepsy-like model. KA injection (single IP injection of 10 mg/Kg) led to the transfer of Cre mRNA to the brain, indicated by marker gene expression in neurons as well as in microglia (S3 Fig). Recombination levels in DA neurons were similar to those seen in DG granule neurons in the HC but much lower in microglia compared to the LPS model. Altogether, levels of marker gene expression were comparable to those after LPS injection (NeuN: 18.5% ± 14.6% SD, *n* = 5), although with a higher variability, as well as a shift to more neuronal and less microglial recombination in the HC (Iba1: 35.2% ± 16.3% SD, *n* = 5) (S3 Fig). Variations in the SN were less pronounced (S3 Fig) (TH: 23.2% ± 3.1% SD; Iba1 29.2% ± 7.0%, *n* = 5).

Next, we wanted to control for the possibility of immune cell infiltration from the periphery caused by the injection of LPS or KA. Peripheral macrophages can be distinguished from brain resident microglia by their expression of the integrin subunit alpha 4 (Itga4/CD49d) [11]. Immunohistochemical staining of brain sections (*n* = 3 mice for each condition) showed no observable CD49d-positive cells in the brain parenchyma, thereby ruling out this possibility (S4A to S4E Fig). Conditions like stroke or glioblastoma lead to high infiltration of peripheral monocytes and were used as positive controls (S4F and S4G Fig).

The presence of both the Cre-expressing and Cre reporter construct in the same cells leaves the theoretical possibility of marker gene induction being caused by unspecific expression of Cre recombinase. To address this issue, we injected KA into the HC of one hemisphere of ROSA26-EYFP Cre reporter mice (*n* = 3), which lack endogenous Cre expression, and saline solution into the contralateral side. Subsequently, we injected EVs isolated from the plasma of LPS-stimulated vav-iCre mice into the tail vein (experimental scheme in S5A Fig). Analysis of brains after 48 h showed recombination events only at the site of KA injection but not in the contralateral hemisphere (S5B and S5C Fig). Thus, recombination could have only been induced by EVs entering the brain via the peripheral circulation. Furthermore, local neuronal activation by KA injection was necessary and sufficient for EV uptake, in contrast to the injection of the carrier solution alone. Intracranial injection of KA together with EVs isolated from vav-iCre mouse plasma into ROSA26-EYFP mice (*n* = 3) led to more widespread marker gene expression (S5D Fig) compared to that observed after peripheral injection of Cre-containing EVs. Intracranial injection of KA together with EVs from wild-type animals (*n* = 3) did not lead to the expression of the recombination marker (S5E Fig), ruling out unspecific labeling caused by KA-induced cell death.

Overall, we interpreted these results as an indication that neuronal activity may be a factor regulating EV uptake and turned to experimental paradigms suitable to specifically address this issue.

### Inhibition of the ubiquitin–proteasome system leads to specific EV uptake in DA neurons

**Fig 2:**
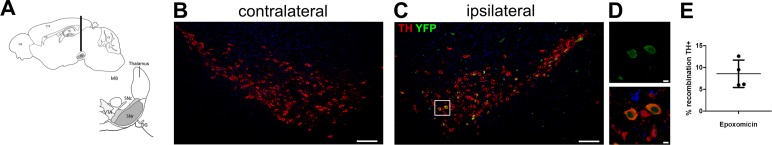
Neuronal activation by proteasome inhibition is sufficient to trigger EV uptake in DA neurons. (A) Unilateral infusion of the selective proteasome inhibitor epoxomicin into the ventral midbrain leads to increased in vivo firing frequencies of DA SN neurons. Two weeks after infusion, no marker-gene–expressing DA neurons were discernible in the contralateral hemisphere (B). In contrast, DA neurons in the ipsilateral hemisphere frequently initiated marker gene expression (C and D). (E) Percentages of marker-positive DA neurons in the SN (average of *n* = 4 ± SD. *p* = 0.0571 two-tailed nonparametric Wilcoxon–Mann–Whitney U test compared to zero marker-gene–positive cells in control animals, *n* = 3). Underlying data can be found in S2 Table. Scale bars, 100 μm in B, C; 10 μm in D. CC, corpus callosum; CE, cerebellum; CTX, cortex; DA, dopaminergic; DG, dentate gyrus; EV, extracellular vesicle; HC, hippocampus; LV, lateral ventricle; MB, midbrain; OB, olfactory bulb; SN, substantia nigra; SNc, substantia nigra pars compacta; SNr, substantia nigra pars reticulata; TH, tyrosine hydroxylase; VTA, ventral tegmental area; YFP, yellow fluorescent protein.

Transient increases in neuronal firing are a common pathological feature of Parkinson’s disease and well described in animal models in which protein degradation via the ubiquitin–proteasome system is impaired either genetically or pharmacologically [12,13]. We unilaterally infused the selective proteasome inhibitor epoxomicin [14] into the ventral midbrain as described previously [13] (Fig 2A). Two weeks after infusion, mice were killed, and the brains were processed for quantitation of EYFP-expressing DA neurons in the SN. We observed no recombination events in TH-positive neurons in the contralateral site of infusion and no recombination in animals that received an infusion of carrier solution (*n* = 3) (Fig 2B). In the ipsilateral site of infusion, we could detect EYFP-TH double-positive neurons (Fig 2C to 2E) (8.6% ± 3.1% SD, *n* = 4). These results are in line with a highly specific uptake of EVs in DA neurons when firing frequencies in DA SN neurons are increased. Interestingly, we could detect none to only very few recombined microglia, which may be due to the reported anti-inflammatory properties of epoxomicin [14].

### Specific induction of neuronal activity by optogenetic methods

**Fig 3:**
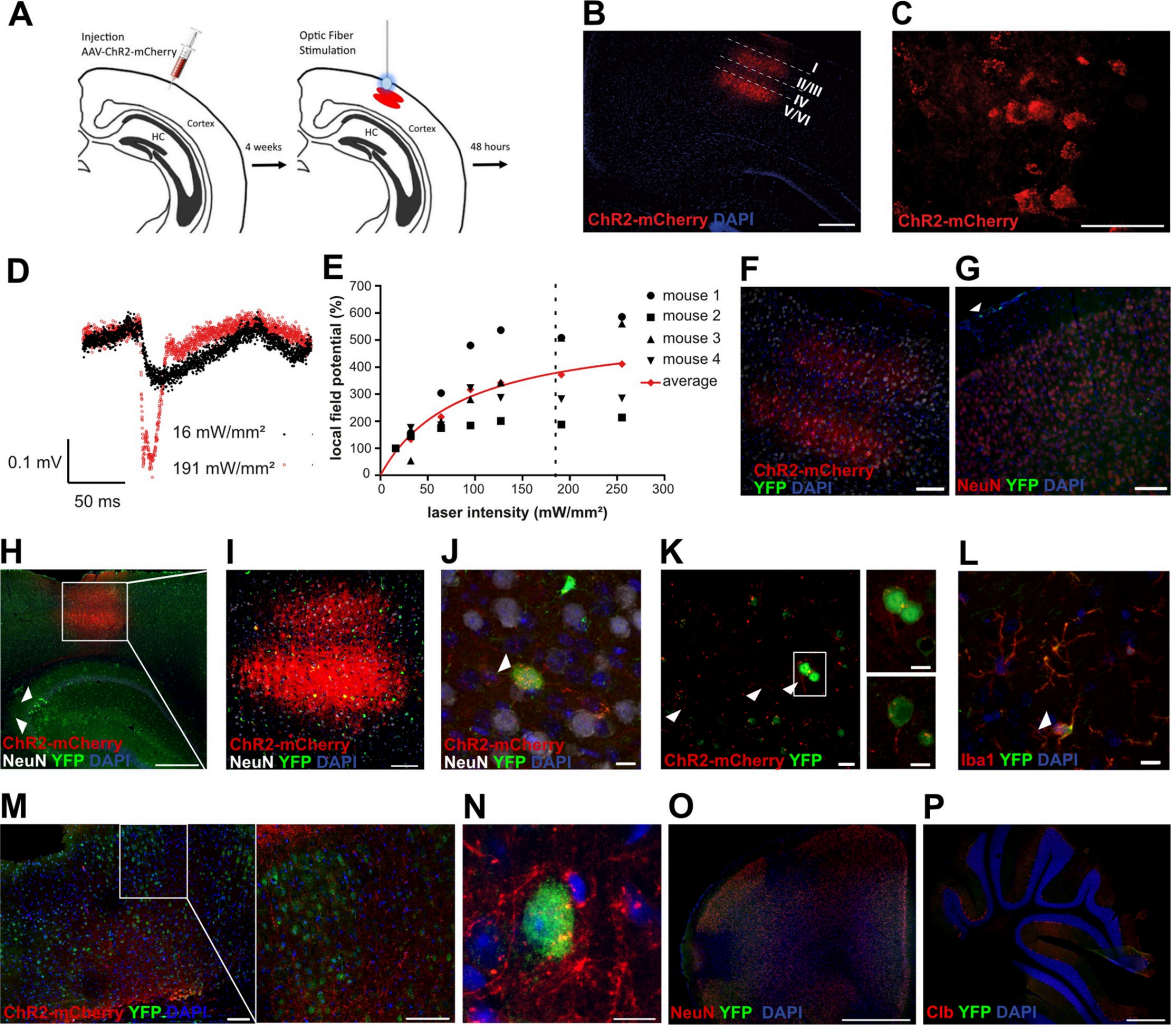
Neuronal activation by optogenetic stimulation. (A) Scheme for the injection of ChR2-mCherry-AAV in layer 5 of the primary visual cortex and optical stimulation. (B) Expression pattern of ChR2-mCherry in primary visual cortex (V1) 4 weeks after AAV injection. Layer-specific expression of ChR2-mCherry, mainly in layer V of V1. (C) Membrane-bound expression of ChR2-mCherry. (D) Representative in vivo LFP traces from mouse 4 upon optic-fiber–based illumination with blue light with an intensity of 16 and 191 mW/mm^2^ at the tip of the fiber. (E) Normalized LFP amplitudes plotted against light intensity. Averaged amplitude was fitted into a nonlinear curve. The dotted line marks the light intensity used in subsequent experiments (approximately 185 mW/mm^2^). Underlying data can be found in S2 Table. (F) Control sections of AAV-injected mice without optical stimulation as well as from mice that received only an optical stimulation without AAV injection (G) (the white arrowhead indicates marker-gene–positive meningeal macrophages). (H) Recombination occurs in ChR2-mCherry–positive and negative neurons and also in the HC (arrowheads) (H–K) but only in a few Iba1-positive microglia (L). Projections of ChR2-expressing neurons extend to the contralateral side and recombination can also be observed in target areas (M + N). No marker-gene–positive cells can be observed in sections from the forebrain (O) or the cerebellum (P). Scale bars, 500 μm in B, H, P; 50 μm in C; 100 μm in F, G, I, M; 10 μm in J, N; 5 μm in L; 20 μm in K; 1 mm in O. AAV, Adeno-Associated Virus; ChR2, channelrhodopsin-2; Clb, calbindin; HC, hippocampus; LFP, local field potential; YFP, yellow fluorescent protein.

To directly stimulate neuronal activity with high specificity, we turned to an optogenetic approach [12]. Channelrhodopsin-2 (ChR2) is a rapidly gated blue-light–sensitive cation channel leading to membrane depolarization that can evoke action potential firing [15] in neurons. We injected Adeno-Associated Virus (AAV) encoding ChR2 fused with mCherry under an excitatory-neuron–specific CamKIIA promoter into the primary visual cortex of vav-iCre-ROSA26-EYFP mice (experimental scheme in Fig 3A). We used the primary visual cortex because it is not compartmentalized like the barrel cortex, which would hinder free diffusion of EVs, as well as because of our long-standing expertise in both imaging and optogenetic modulating network activity in this region. We first assessed the expression profile of the opsin ChR2 fused with the fluorophore mCherry. We found strong and membrane-bound expression of ChR2-mCherry mainly in layer V and, to a lesser extent, in layers II and III (Fig 3B and 3C). We next tested the functionality of our optogenetic approach by conducting in vivo electrophysiological field potential recordings. For that, we illuminated the opsin-expressing region with an optic fiber emitting blue light of varying light intensities 4 weeks after rAAV2-CaMKIIa-hChR2-(H134R)-mCherry-Woodchuck Hepatitis Virus Posttranscriptional Regulatory Element (WPRE)-polyadenylation site (pA) virus injection. We found a typical sigmoidal dose–response curve [16] (Fig 3D and 3E). Based on these data, we chose the light intensity for subsequent experiments that reliably evoked strong neuronal responses. To control for the unspecific induction of EV uptake by the experimental procedure, we also analyzed brains from animals that received an AAV injection without optical stimulation (*n* = 4) (Fig 3F) or for whom we performed an optical stimulation without prior injection of AAV (*n* = 4) (Fig 3G). In both cases, the treatment did not induce any neuronal recombination events. In contrast, in all animals with a combination of an AAV injection and optical stimulation (*n* = 4), we observed EYFP- and NeuN-positive neurons (Fig 3H to 3K). The average number of marker-gene–positive cells in the area of ChR2-mCherry expression indicated by the white box in Fig 3H was 43.55 cells per 0.01 mm^3^ (±6.82 cells per 0.01 mm^3^ SD; average of 3 animals) with 3.7% (±3.5% SD) of EYFP-positive cells being microglia (Fig 3L) (*p* = 0.00909 two-tailed nonparametric Wilcoxon–Mann–Whitney U test compared to zero marker-gene–positive cells in control animals, *n* = 9). However, only a part of recombined neurons coexpressed ChR2-mCherry, and most recombination events were actually outside the injection area in adjacent cortical areas and in the HC (Fig 3H to 3K). Indeed, virally transduced neurons send projections to the contralateral hemisphere with accompanying recombination in the surrounding area (Fig 3M and 3N). We interpret this as a network stimulation effect whereby connected neurons will increase firing, thus leading to the uptake of EVs in cells that were not directly stimulated. We did not observe any recombination events in areas distant from the stimulation site such as the forebrain and cerebellum (Fig 3O and 3P), underlining the highly localized stimulation.

### Hippocampal neuronal activity induced by novel object placement

**Fig 4:**
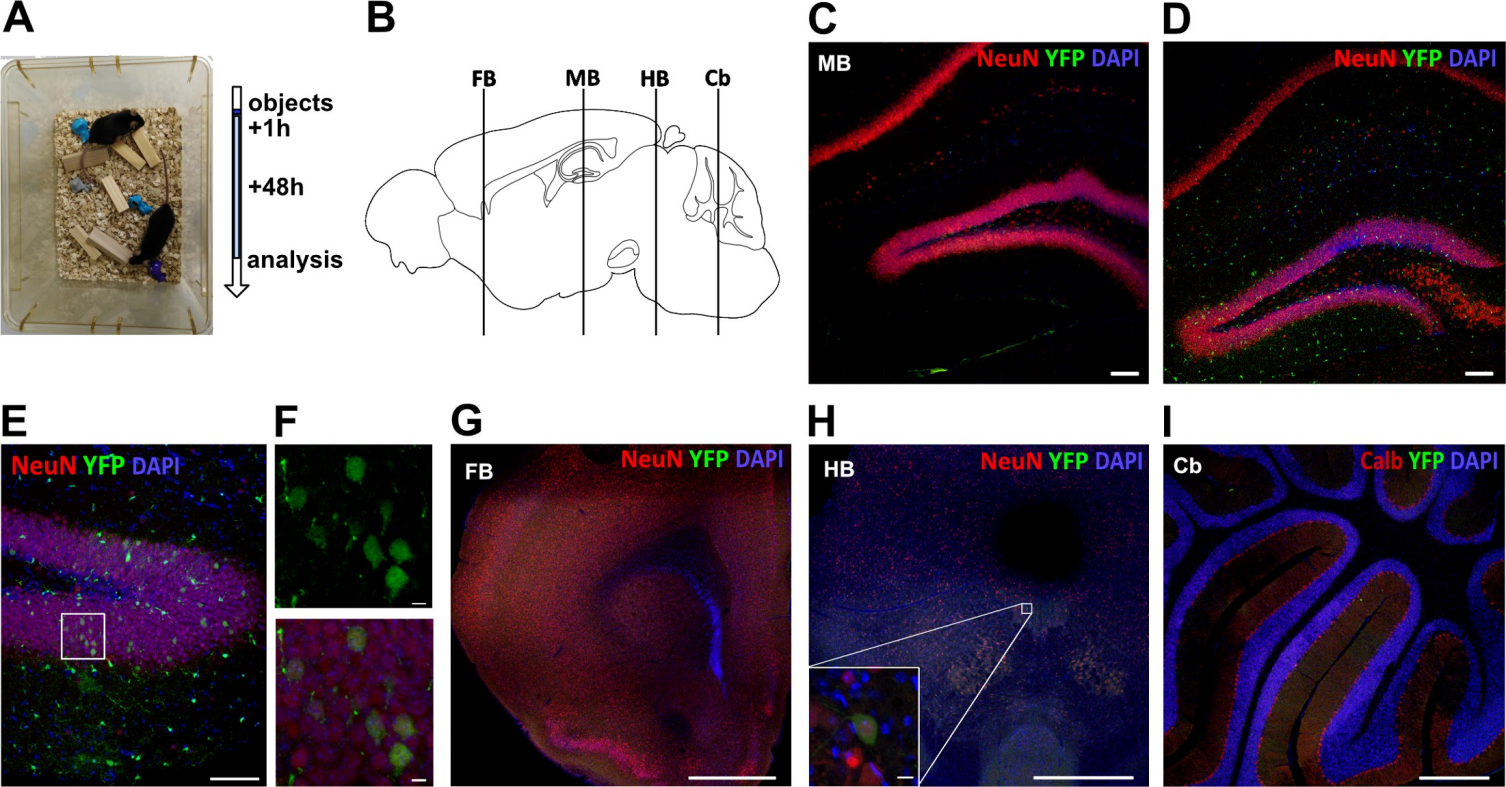
Neuronal stimulation by novel object placement. (A) Novel objects were placed in mouse cages and left for 1 hour. Brains were analyzed along their entire rostrocaudal length 48 h thereafter (B). No marker gene expression was observed in controls in contrast to placement mice (C). Recombination occurred mainly in neurons in hippocampal areas DG and CA1 and CA2 but also in some microglia (D–F). Marker gene expression was absent or very rare in other brain areas such as forebrain (G), hindbrain (H), and cerebellum (I). A single marker-gene–positive neuron in the hindbrain is shown in inset (H). Scale bars, 100 μm in C, D, E; 500 μm in H, I; 10 μm in F and inset in H; 1 mm in G. Calb, calbindin; Cb, cerebellum; DG, dentate gyrus; FB, forebrain; HB, hindbrain; MB, midbrain; YFP, yellow fluorescent protein.

So far, in our experiments, we triggered EV uptake via pathological events or ectopic stimuli. Next, we probed whether increased neuronal activity induced by behavioral paradigms would suffice for neuronal EV uptake. To this end, we chose the induction of hippocampal activity by environmental novelty. The HC is involved in the formation of contextual memory, and it has been shown that the addition of novel objects into a mouse cage leads to a selective increase in neuronal c-fos (an immediate early gene) expression in the HC [17]. We placed several new objects into a cage with 1–2 vav-iCre-ROSA26-EYFP mice. The objects were removed after one hour (Fig 4A). After 48 h, animals were killed, and their brains were analyzed at different rostrocaudal levels (Fig 4B). We could not detect marker-gene–positive neurons in animals that were housed in cages that were left unchanged (*n* = 5) (Fig 4C). In the animals from the cages with new objects (*n* = 9), we observed EYFP-positive neurons in the HC (Fig 4D to 4F) in all but one animal. We counted marker-gene–positive cells at an average frequency of 117 cells per 0.01 mm^3^ (±14.1 cells per 0.01 mm^3^ SD, *n* = 3) for the DG and 45 cells per 0.01 mm^3^ (±13.8 cells per 0.01 mm^3^ SD, *n* = 3) for the CA1 and CA2 hippocampal subfields, with 13.7% (±2.1 SD, *n* = 3) of EYFP-positive cells being microglia (Fig 4F) (*p* = 0.0357 two-tailed nonparametric Wilcoxon–Mann–Whitney U test compared to zero marker-gene–positive cells in control animals, *n* = 5). Underlying data can be found in S2 Table. We did not, or only very rarely, detect any EYFP-positive neurons in the forebrain (Fig 4G), hindbrain (Fig 4H), or cerebellum (Fig 4I), where no activation would be expected.

## Discussion

In summary, we demonstrate that functional transfer of molecules contained in EVs that are released from hematopoietic cells and taken up by neurons is widespread and consistent with the notion that EV uptake is triggered by neuronal activity, both under pathological and physiological conditions.

Conceptually, in the experimental settings we used, EV signaling appears to follow more of a “demand–pull” rather than a “supply–push” principle, in that the status of the target cell determines uptake rather than the availability of EVs. Surprisingly, EVs could overcome the BBB, even though some of the conditions we used are not known to damage the BBB and we never observed infiltrating peripheral blood cells. This could indicate that EVs enter into the brain via transcytosis, similar to recent observations in a tumor model [18]. Furthermore, neuronal activity that leads to the uptake of EVs might simultaneously regulate BBB permeability, as shown previously [19]. This presents an interesting extension to the topic of activity-dependent neurovascular coupling.

For neurodegenerative diseases like Parkinson’s disease, research in the EV field focuses mainly on the intercellular transfer of misfolded proteins or the use of EVs as diagnostic markers for neurodegenerative processes [20,21]. In particular, EVs are considered as a possibility to either clear or transfer alpha-synuclein from or between cells. Our results now suggest an ongoing role of EVs that may impact disease onset and progression. We do not know yet whether EV uptake is beneficial or detrimental for neurons and whether the cell of origin determines its function. Notably, oligodendrocyte-derived EVs can have trophic effects on neurons in vitro [22], and macrophage-derived exosomes containing functional NADPH oxidase 2 complexes promote axonal regeneration [23]. On the other hand, neuronal activity has been linked to tau propagation [24], and uptake of EVs may at least in part explain this transneuronal spread.

The surprising fact that we observe EV uptake under physiological conditions induced by neuronal activity due to an enriched environment suggests a role of EV signaling going beyond a reaction to pathology-induced cellular changes. This opens the question of whether cognitive processes or behavior can be influenced by EVs originating from the outside of the central nervous system in the medium or long term.

While we use multiple experimental models that converge on the notion that neuronal activity is a stimulus that triggers uptake of EVs from the circulation, some methodological shortcomings need to be kept in mind. Currently, it is not possible to specifically modulate or block EV release, particularly not in vivo, which is a general drawback in the field. In addition, as a formal proof, it would be desirable to combine global neuronal stimulation with a complete blockage of electrical activity in local populations of neurons. While this could be achieved in an in vitro setting using cell or slice cultures, this would in turn introduce artifacts regarding the biology of EVs caused by their extraction and purification, as well as the likely altered behavior of neural cells with regard to the regulation of EV uptake.

In terms of a global communication between the brain and periphery, we have to consider that our transgenic animal model reports EV transfer from peripheral blood populations only, while EVs released from any other organ go unreported. Thus, the extent of the influence of EVs on processes in the brain may be much more substantial than what we observe. Lastly, EVs show great promise in delivering drugs or even RNA across the BBB, and efforts are being made to target these to specific cellular populations. Our results show that, at least in the case of neurons, EVs seem to naturally target cells that are stimulated both by pathological and physiological processes with high specificity.

## Materials and methods

### Ethics statement

All experiments were performed in compliance with the German law on animal experimentation, following the guidelines of the European Union for the use of animals in research (European Union Directive 2010/63/EU), and were approved by the regional Ethical Commission for Animal Experimentation of the state of Hessen (ethical permissions Gen. Nr. F90/10, F94/12, FK/1090, F94/19) and the state of Rhineland-Palatina (ethical permission G 14-1-040).

Additional animal experiments were conducted according to the guidelines in the "Guide for the Care and Use of Laboratory Animals" of the National Research Council (1996) and according to National Institute of Health guidelines and were approved by the Institutional Animal Care and Use Committee for Massachusetts General Hospital, Boston, USA, as required by the Public Health Service (PHS) Policy on Humane Care and Use of Laboratory Animals.

### Animals

All mice were group housed (maximum of 4 mice per cage) under standard laboratory conditions with a 12:12 h light/dark cycle, with food and water provided ad libitum. All experiments were performed on 6- to 30-week–old mice during the day cycle. Cage enrichment experiments were conducted between 10:00 and 13:00 during the day cycle. Both male and female mice were used because we never observed any obvious differences between the sexes, including in our earlier studies. The vav-iCre mice were a gift by Dimitris Kioussis [7] (JAX-mice stock number 008610). The vav-iCre line was cross-bred with the Cre reporter mice ROSA26-EYFP (JAX-mice stock number 005130). For the detection of double-transgenic offspring, a drop of blood was collected from each pup and processed for analysis by flow cytometry (BD FACS Canto II Flow Cytometer, BD Biosciences, San Jose, CA, USA) for EYFP expression. The EpoR-iCre mice were obtained from Dr. Stuart Orkin (Boston Children’s Hospital, Boston, MA, USA) and are deposited in the Jackson Laboratories; these were cross-bred with the ROSA26 mTom/mGFP mice for the experiments.

### EV preparation

Peripheral blood from deeply anesthetized reporter or double-transgenic mouse previously injected with LPS was collected during perfusion. Platelet-free plasma was isolated through centrifugation steps (Heraeus Labofuge 400R; Thermo Fisher Scientific, Waltham, MA, USA) of 2,500 rpm for 15 min at RT twice and diluted 1:1 with 10 mM HEPES buffer (A6916,0125; AppliChem GmbH, Darmstadt, Germany) in Millipore water (EMD Millipore, Burlington, MA, USA). The solution was 0.45-μm filtered to remove larger particles and mixed with polyethylene glycol (PEG) 8000 (Rotipuran, 0263.1, Roth) solution 1:5 for precipitating vesicles during an overnight incubation at 4°C. Samples were centrifuged at 1,500 × *g* for 30 min at 4°C, and pellets were resuspended in 10 mM HEPES buffer for an ultracentrifugation step (Sorvall WX Ultra Series 80; Thermo Fisher Scientific) at 100,000 × *g* for 90 min. The pellets were resuspended in 10 mM HEPES buffer and kept at 4°C for immediate use or at −80°C for longer storage.

### In vivo experiments: Stereotactic surgeries

For experiments using stereotactic surgeries for viral injections and optic fibers, mice were anesthetized with isoflurane (Floren, Abbott Laboratories, Lake Bluff, IL, USA), 2% v/v in O_2_ and placed in a stereotaxic apparatus (Model 900/940, KOPF Instruments, Tujunga, CA, USA) with adapted components to allow mouse inhalation anesthesia. Before surgery, Xylocain (AstraZeneca, Cambridge, UK) was administered as a local analgesic at the incision site on the skin. For closing incisions, a few drops of the tissue adhesive Surgibond (190740, SMI sutures; Praxisdienst, Longuich, Germany) were used.

For the optogenetics assay, AAV (titer of 10^13^ genomic copies per ml) with CamKII promoter-driven hChR2(H134R)-Cherry (VB4411; Vector Biolabs, Malvern, PA, USA) was used. Intracranial injection was performed unilaterally with elongated glass capillaries (612–2401, Hirschmann; VWR International, Radnor, PA, USA) at a 35° angle, and the injected volume was 1 μl (coordinates in millimeters from bregma: M/L = 2.5; A/P = −3.1; D/V = 0.5). At the end of the infusion, needles were kept at the site for 4 min and then slowly withdrawn. Viral expression was assessed 4 weeks after surgery.

For light stimulation, a 200-μm multimode fiber with a numerical aperture of 0.39 (Thorlabs, Munich, Germany) was positioned unilaterally (touching the cortex) over the site of the AAV injection. The fiber was coupled to a solid-state 488-nm 50-mW laser (Lighthub, Rodgau-Dudenhofen, Germany) controlled by the omicron proprietary software. The laser output power was adjusted to read 5.8 mW measured at the fiber (approximately 185 mW/mm^2^). The laser was pulsed at 10 Hz with 10-ms pulse width at 0.1 Hz for 1 h using an external pulse stimulator (CED 1401 Cambridge Electronic Design, Cambridge, UK). After the stereotaxic surgery, the animals were left for 2 days to allow for the expression of EYFP and then perfused for further analysis.

Local field potential (LFP) recordings in vivo were performed using glass pipettes (impedance approximately 1 MΩ) in layer V of primary visual cortex 4 weeks after rAAV2-CaMKIIa-hChR2-(H134R)-mCherry-WPRE-pA virus injection. Mice were anesthetized with isoflurane by inhalation and placed on a warming pad (37°C). A silver wire was inserted into the cerebellum as the ground electrode. To induce neuronal activity, single blue-light pulses (10 ms) were administered at 0.1 Hz via a 200-μm–diameter multimode fiber. The laser output power at the fiber tip was adjusted to 0.5, 1, 2, 3, 4, 6, and 8 mW (corresponding to 16, 32, 64, 95, 127, 191, and 255 mW/mm^2^ at fiber tip). At each intensity, 10 sweeps were recorded and averaged. In Fig 3E, LFP amplitudes were normalized by the amplitudes evoked at 16 mW/mm^2^ intensity.

To induce peripheral inflammation, 200 μl of LPS (1 mg/Kg LPS from *Escherichia coli* O55:B5, L2637; Sigma-Aldrich, St. Louis, MO) was injected IP every 24 h for 2 days (2 injections in total). After the last injection, mice were kept for 48 h; then animals were perfused for further analysis. For KA injection, 100 μl of KA (2.5 mg/ml, ab120100; Abcam, Cambridge, UK) was IP injected once. After 48 h, mice were killed and transcardially perfused for further analysis.

For epoxomicin infusion, a craniotomy was performed at the following coordinates in millimeters from bregma: M/L = 0.9, A/P = −2.85, D/V = 4, and 10 μM epoxomicin in 1% DMSO or 1% DMSO alone as control were infused unilaterally using a micropump (UMP3-1; WPI, Berlin, Germany; 10-lL nanofil syringe, 33-gauge steel needle; flow rate of 100 nL/min). After a 2-week postinfusion period, the anesthetized animals were transcardially perfused for further analysis.

For the combination of IV EV injection and local neuronal stimulation, ROSA26-EYFP mice received an intracranial injection of 0.5 μg of KA in 1 μl of saline solution (ipsilateral) and 1 μl of saline solution only (contralateral) at the coordinates in millimeters from bregma: M/L = ±1.5; A/P = −2; D/V = 2). Directly afterwards, 100 μl of a preparation of EVs from blood plasma of an LPS-injected vav-iCre mouse was IV injected via the tail vein. Mice were left for 48 h to allow for reporter gene expression and were transcardially perfused for further analysis.

### Novel object placement

Cages used for the experiment were standard cages for mice (365 × 207 × 140 mm, 1284L Eurostandard Type II L). One or two mice continuously inhabited this cage before an assortment of objects of different sizes and colors were placed in the cage for 1 hour before they were removed again. Mice were kept for an additional 2 days before they were killed, and brains were removed for further analysis.

### Tissue processing

At the end of the experiment, mice were killed with an overdose of Ketavet IP (100 mg/kg)/Xylacin (5 mg/kg) and transcardially perfused with PBS followed by 4% cold PFA in PBS, and brains were removed for subsequent analysis. All conserved organs were postfixed in 4% PFA in PBS for 24 h. For cryosectioning, organs were cryoprotected in 15% sucrose for an additional 24 h before they were embedded and serially sectioned (10 μm) as a frozen block, or tissue was snap-frozen in methyl butane previously cooled in liquid nitrogen. Sections were attached to glass slides and stored at −20°C until further use. For fresh fixed tissue, serial coronal sections (30 μm) were cut on a vibratome (Leica VT1000S; Leica, Wetzlar, Germany) and kept in PBS at 4°C until further use.

### Antibodies

Primary antibodies used in the study were rabbit Calbindin D28-k (Swant, CB-38, 1:5,000), rat DA transporter DAT (Millipore, MAB369, 1:1,000), rabbit glial fibrillary acidic protein GFAP (DAKO, ZO334, 1:1,000), chicken GFP (Abcam, ab13970, 1:500), rabbit Iba1 (WAKO, 019–19741, 1:1,000), mouse NeuN (Millipore, MAB377, 1:300), rat anti-mouse CD49d, Clone R1-2 (RUO) (BD Horizon, 1:400), mouse tyrosine hydroxylase TH (Millipore, MAB318, 1:1,000), rat anti-mouse CD31 (BD Pharmingen 557355, 1:100), and mouse Olig2 (Millipore MABN50, clone 211F1.1, 1:100).

Secondary antibodies used were Alexa Fluor 647 goat anti-rat (Life Technologies, A21247, 1:1,000; Carlsbad, CA, USA), Alexa Fluor 488 goat anti-chicken (Abcam, ab150169, 1:1,000), Alexa Fluor 568 goat anti-mouse (Invitrogen, A11004, 1:1,000; Carlsbad, CA, USA), Alexa Fluor 647 goat anti-mouse (Invitrogen, A21235, 1:1,000), Alexa Fluor 568 goat anti-rabbit (Invitrogen, A11011, 1:1,000), and Alexa Fluor 647 goat anti-rabbit (Invitrogen, A21244, 1:1,000).

### Immunohistochemistry

For immunofluorescence staining, brain slices were permeabilized with PBS-Triton X-100 0,1%, blocked in 10% NGS, and incubated overnight at 4°C with the primary antibody. On the next day, slices were washed and incubated with the secondary antibody for 2 h at 4°C. After a final wash, brain slices were stained with DAPI (Sigma-Aldrich, D9542, 1:1,000) and mounted with Aqua-Poly/Mount (18606–20; Polysciences, Warrington, PA, USA).

For light microscopy, 12-μm cryosections were stained on a fully automated Leica Bond III (Leica Biosystems) using the Leica Bond Polymer Refine Detection Kits (DS9800 + DS9390).

### Image acquisition

Images were captured with either an epifluorescence microscope (Nikon Eclipse 80i; Nikon, Tokyo, Japan) or a confocal inverted microscope (Nikon Eclipse TE2000-E). For confocal imaging, a z-stack of pictures of areas of interest was obtained using different picture size magnifications. Images were analyzed with NIS-elements imaging software (version 4.13.05) and ImageJ (https://imagej.nih.gov/ij/). To assess EYFP and mCherry expression in the contralateral hemisphere, we used a confocal microscope (SP8, Leica, Mannheim, Germany) using multiple objectives at 20×, 40×, and 63× objective (HCX PL APO 20×/0.7 dry, HC PL APO 40×/1.40 Oil CS2, HC PL APO 63×/1.40 Oil CS2; Leica).

### Statistical analyses

No statistical methods were used to predetermine sample sizes. Sample sizes were chosen based on data from previous experience (i.e., no observable EV-mediated Cre transfer in control animals). Animals were randomly allocated into different experimental groups without use of specific randomization. Mice showing incorrect injection sites or optic fiber placement were excluded. A list of all animals used can be found in S1 Table. Because the value of the number count of marker-gene–positive cells for each animal of the control group is zero, variability is strongly different between the groups, and the standard *t* test could not be used. Furthermore, normal distribution could not be tested effectively. Therefore, a two-sided nonparametric Wilcoxon–Mann–Whitney U test was performed to compare the outcomes between the 2 groups. It should be emphasized that the Wilcoxon–Mann–Whitney U test is a ranked-order test that ignores the high absolute differences between experimental and control group (all zero) of this study. To further characterize the outcomes, mean and standard deviation and additionally median, minimum, and maximum were presented. If at least 5 observations are available, 90% confidence intervals for median values were calculated. The computations of the statistical values can be found in S2 Table.

## Supporting information

S1 Fig Analysis of marker gene expression after LPS-induced inflammation in multiple brain areas. LPS, lipopolysaccharide.
(TIFF)

S2 Fig Absence of marker gene expression in multiple lineages. Costaining for marker gene expression and lineage markers for astrocytes (GFAP, A and B), oligodendrocytes (Olig2, C and D), and endothelial cells (CD31, E and F) showed no costaining except in a single endothelial cell (F). White arrowheads indicate an endothelial cell coexpressing EYFP, and white frames delineate the area displayed in the magnification images. Scale bar A, C, E, 100 μm; B, D, F, 20 μm. EYFP, enhanced yellow fluorescent protein; GFAP, glial fibrillary acidic protein.
(TIFF)

S3 Fig Recombination in HC and SN after KA injection. (A) HC with DG showing recombination in neuronal and non-neuronal cells. (B) Magnified view from another area of the DG with marker-gene–expressing granule neurons as well as Iba1-positive microglia in (C). (D) SN with TH-positive but also TH-negative neurons expressing EYFP as well as microglia in (E). (F) Percentages of marker-gene–positive neurons or microglia in the HC or SN. Data are presented as mean ± SD, *n* = 5. *p* = 0.000666 two-tailed nonparametric Wilcoxon–Mann–Whitney U test for all populations compared to zero marker-gene–positive cells in all control animals, *n* = 10. Underlying data can be found in S2 Table. Scale bars, 100 μm A, D; 10 μm E; 20 μm B, C. DG, dentate gyrus; EYFP, enhanced yellow fluorescent protein; HC, hippocampus; KA, kainate; SN, substantia nigra; TH, tyrosine hydroxylase.
(TIFF)

S4 Fig LPS or KA injection does not lead to the infiltration of peripheral blood macrophages to the brain parenchyma. All sections were stained with CD49d and counterstained with HE. Brain sections from LPS-injected mice (*n* = 3) do not show CD49d immunoreactive cells in the HC (A) or other brain areas such as cortex (B), except for meningeal macrophages (black arrowheads) (C). (D + E) Likewise, KA injection does not lead to the infiltration of peripheral macrophages (*n* = 3). Arrowheads indicate CD49d-positive choroid plexus cells. In conditions causing a high influx of peripheral blood cells into the brain such as glioblastoma (F) and cerebral ischemia caused by middle cerebral artery occlusion (G), high numbers of CD49d-positive macrophages are visible. Scale bars, 100 μm in A, B, D, E, F, and G; 20 μm in C. HC, hippocampus; HE, hematoxylin–eosin; KA, kainate; LPS, lipopolysaccharide.
(TIFF)

S5 Fig Peripheral injection of Cre-containing EVs leads to marker gene expression after local neuronal stimulation. (A) Experimental scheme for peripheral EV injection followed by local neuronal activation by intracerebral injection of KA with saline solution into the contralateral hemisphere as control. (B) Intracranial KA injection into ROSA26-EYFP mice (*n* = 3) together with IV injection of EVs prepared from the plasma of a vav-iCre mouse leads to induction of marker gene expression in the ipsilateral, but not in the contralateral, side (C). (D) Intracranial injection of KA together with iCre EVs (*n* = 3 mice) led to more widespread YFP expression compared to IV injection of iCre EVs, whereas no YFP-positive cells could be observed after injection of KA into the HC alone (*n* = 3 mice) (E). White frames indicate area of magnification where applicable. Scale bars in B–D: 100 μm right panels, 50 μm in magnified images. EV, extracellular vesicle; HC, hippocampus; KA, kainate; YFP, yellow fluorescent protein.
(TIFF)

S1 Table List of animals used for experiments. (XLSX)

S2 Table Table with all data values and statistical computations. (XLSX)

## Abbreviations

AAV: Adeno-Associated Virus
BBB: blood–brain barrier
ChR2: channelrhodopsin-2
DA: dopaminergic
DG: dentate gyrus
EpoR: erythropoietin receptor
EV: extracellular vesicle
EYFP: enhanced yellow fluorescent protein
GFAP: glial fibrillary acidic protein
HC: hippocampus
IP: intraperitoneal(ly)
Itga4: integrin subunit alpha 4
KA: kainate
LFP: local field potential
LPS: lipopolysaccharide
pA: poly-adenylation site
PEG: polyethylene glycol
PHS: Public Health Service
SN: substantia nigra
TH: tyrosine hydroxylase
WPRE: Woodchuck Hepatitis Virus Posttranscriptional Regulatory Element
YFP: yellow fluorescent protein
