# Supplementary figures and images for: Neuronal activity triggers uptake of hematopoietic extracellular vesicles in vivo
Source: PLoS Biol. 2020 Mar 16;18(3):e3000643. doi: 10.1371/journal.pbio.3000643 (PMC7075544; doi:10.1371/journal.pbio.3000643)

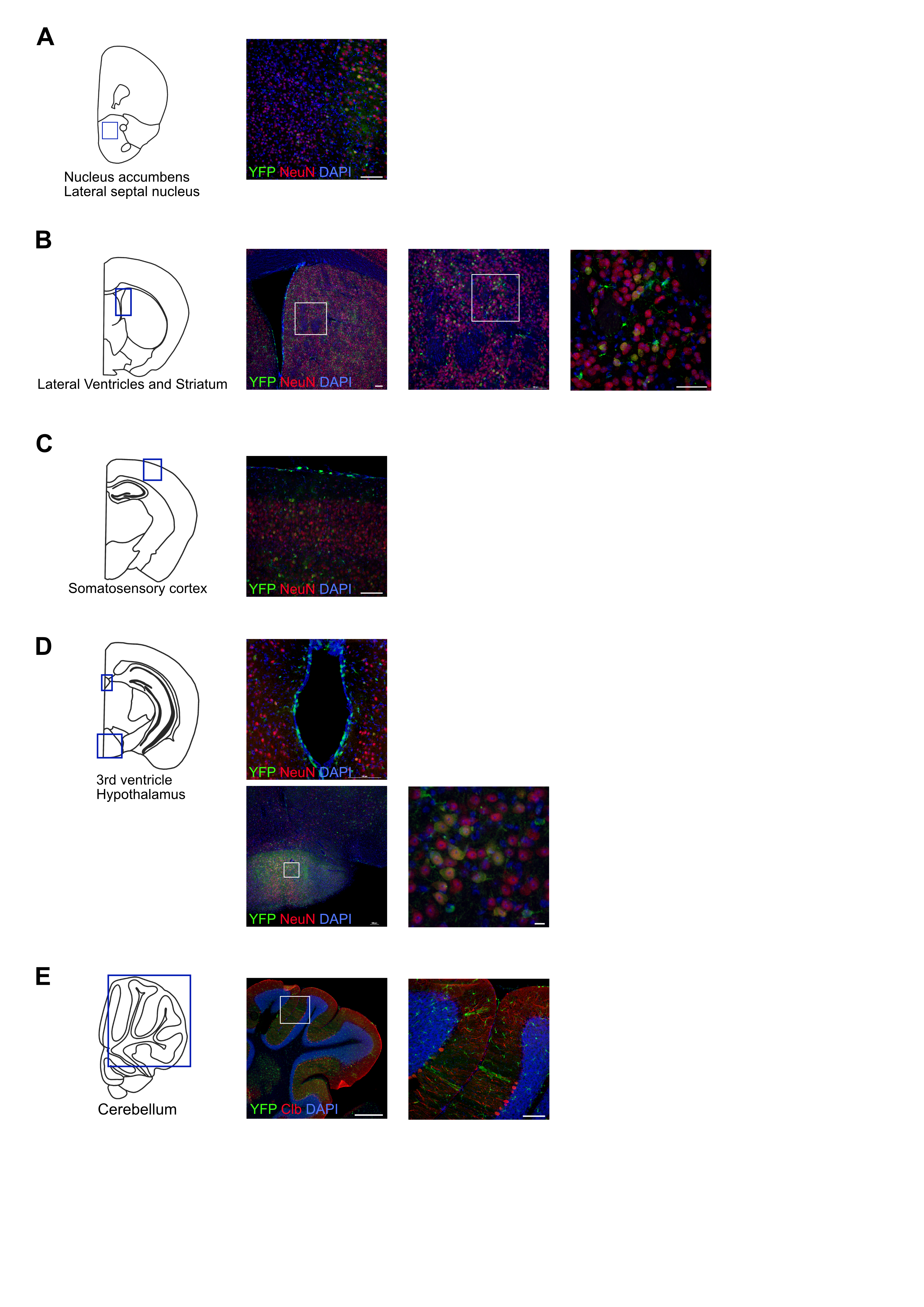

Supplement: S1 Fig — LPS, lipopolysaccharide. (TIFF) [file pbio.3000643.s001.tiff]

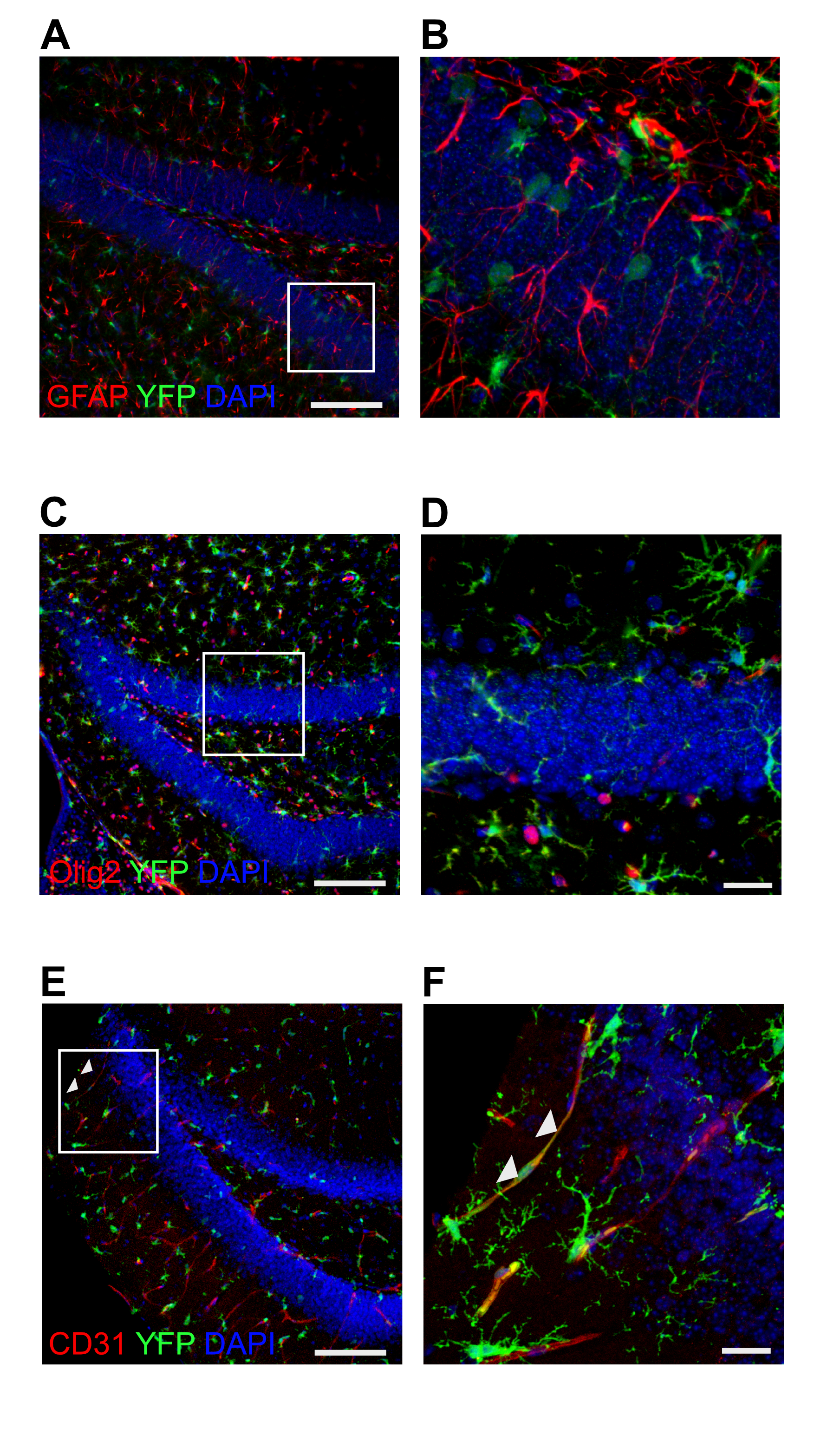

Supplement: S2 Fig — Costaining for marker gene expression and lineage markers for astrocytes (GFAP, A and B), oligodendrocytes (Olig2, C and D), and endothelial cells (CD31, E and F) showed no costaining except in a single endothelial cell (F). White arrowheads indicate an endothelial cell coexpressing EYFP, and white frames delineate the area displayed in the magnification images. Scale bar A, C, E, 100 μm; B, D, F, 20 μm. EYFP, enhanced yellow fluorescent protein; GFAP, glial fibrillary acidic protein. (TIFF) [file pbio.3000643.s002.tiff]

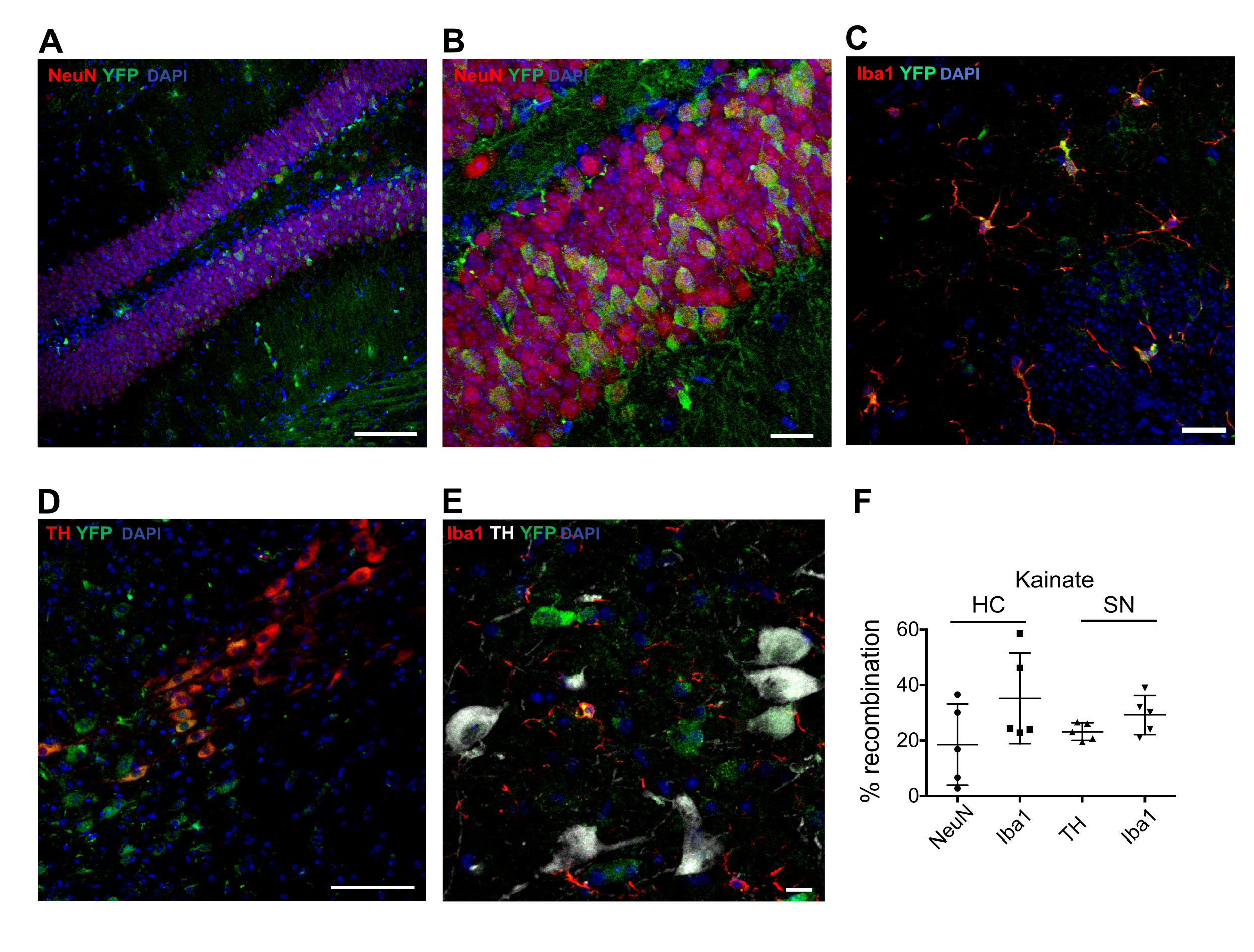

Supplement: S3 Fig — (A) HC with DG showing recombination in neuronal and non-neuronal cells. (B) Magnified view from another area of the DG with marker-gene–expressing granule neurons as well as Iba1-positive microglia in (C). (D) SN with TH-positive but also TH-negative neurons expressing EYFP as well as microglia in (E). (F) Percentages of marker-gene–positive neurons or microglia in the HC or SN. Data are presented as mean ± SD, n = 5. p = 0.000666 two-tailed nonparametric Wilcoxon–Mann–Whitney U test for all populations compared to zero marker-gene–positive cells in all control animals, n = 10. Underlying data can be found in S2 Table. Scale bars, 100 μm A, D; 10 μm E; 20 μm B, C. DG, dentate gyrus; EYFP, enhanced yellow fluorescent protein; HC, hippocampus; KA, kainate; SN, substantia nigra; TH, tyrosine hydroxylase. (TIFF) [file pbio.3000643.s003.tiff]

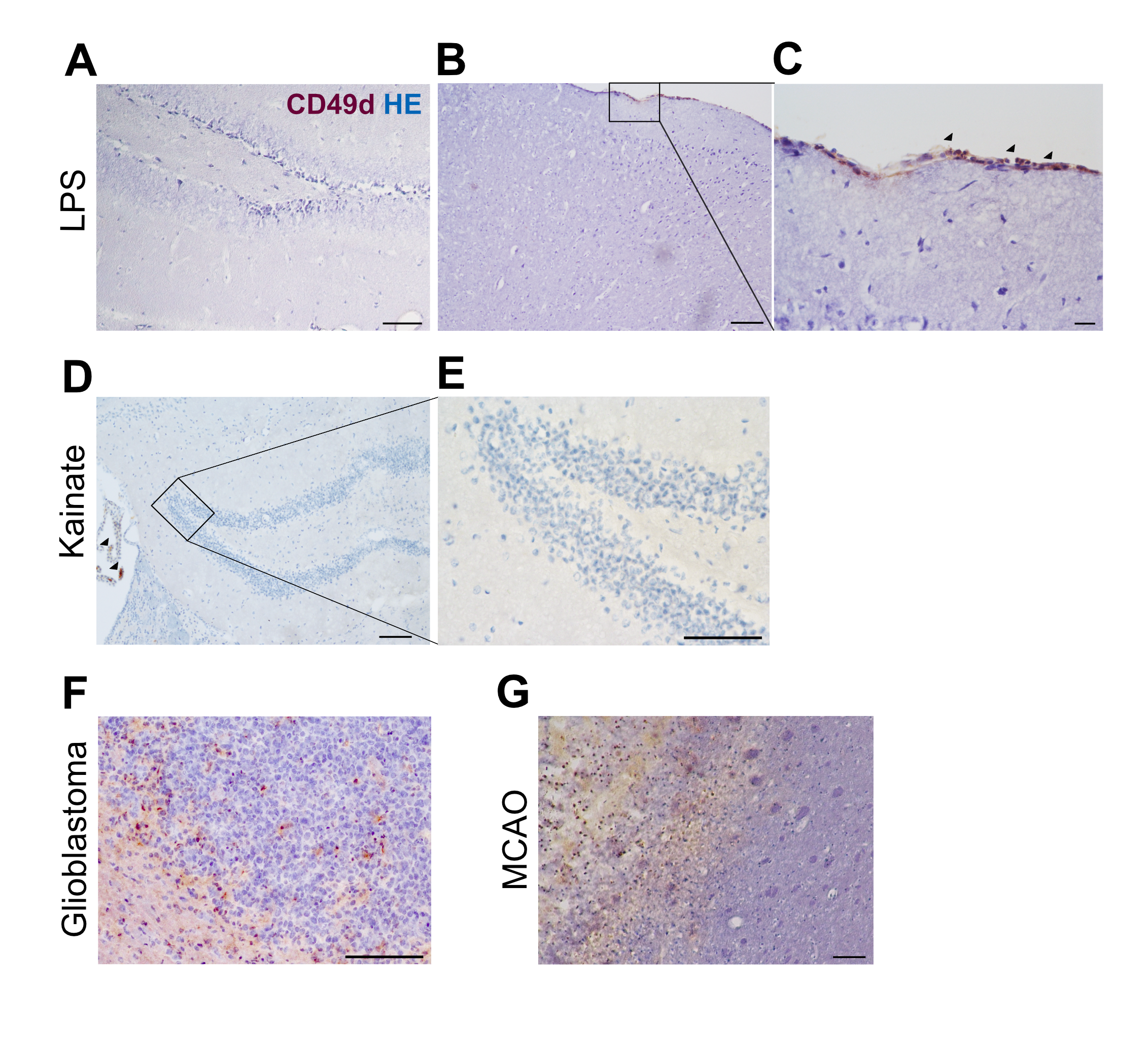

Supplement: S4 Fig — All sections were stained with CD49d and counterstained with HE. Brain sections from LPS-injected mice (n = 3) do not show CD49d immunoreactive cells in the HC (A) or other brain areas such as cortex (B), except for meningeal macrophages (black arrowheads) (C). (D + E) Likewise, KA injection does not lead to the infiltration of peripheral macrophages (n = 3). Arrowheads indicate CD49d-positive choroid plexus cells. In conditions causing a high influx of peripheral blood cells into the brain such as glioblastoma (F) and cerebral ischemia caused by middle cerebral artery occlusion (G), high numbers of CD49d-positive macrophages are visible. Scale bars, 100 μm in A, B, D, E, F, and G; 20 μm in C. HC, hippocampus; HE, hematoxylin–eosin; KA, kainate; LPS, lipopolysaccharide. (TIFF) [file pbio.3000643.s004.tiff]

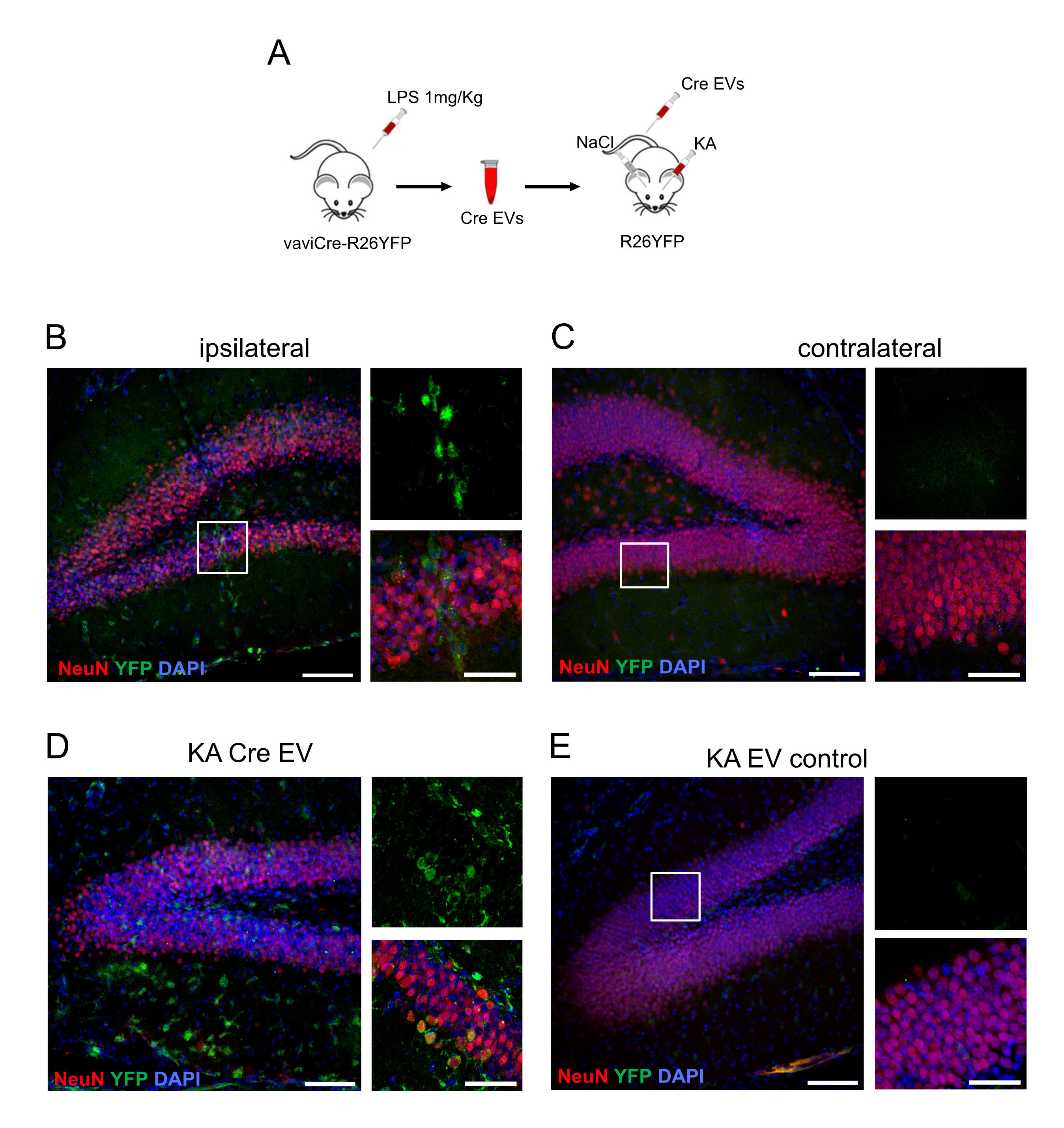

Supplement: S5 Fig — (A) Experimental scheme for peripheral EV injection followed by local neuronal activation by intracerebral injection of KA with saline solution into the contralateral hemisphere as control. (B) Intracranial KA injection into ROSA26-EYFP mice (n = 3) together with IV injection of EVs prepared from the plasma of a vav-iCre mouse leads to induction of marker gene expression in the ipsilateral, but not in the contralateral, side (C). (D) Intracranial injection of KA together with iCre EVs (n = 3 mice) led to more widespread YFP expression compared to IV injection of iCre EVs, whereas no YFP-positive cells could be observed after injection of KA into the HC alone (n = 3 mice) (E). White frames indicate area of magnification where applicable. Scale bars in B–D: 100 μm right panels, 50 μm in magnified images. EV, extracellular vesicle; HC, hippocampus; KA, kainate; YFP, yellow fluorescent protein. (TIFF) [file pbio.3000643.s005.tiff]
